# Whole Exome Sequencing Revealed a Novel GJB1 Pathogenic Variant and a Rare BSCL2 Mutation in Two Iranian Large Pedigrees with Multiple Affected Cases of Charcot-Marie-Tooth

**DOI:** 10.22088/IJMCM.BUMS.8.3.169

**Published:** 2019

**Authors:** Neda Mohsenpour, Hassan Roknizadeh, Mehdi Maghbooli, Majid Changi-Ashtiani, Mohammad Shahrooei, Mansoor Salehi, Mahdiyeh Behnam, Tina Shahani, Alireza Biglari

**Affiliations:** 1 *Department of Genetics and Molecular Medicine, School of Medicine, Zanjan University of Medical Sciences (ZUMS), Zanjan, Iran.*; 2 *Department of Medical Biotechnology, School of Medicine, Zanjan University of Medical Sciences (ZUMS), Zanjan, Iran.*; 3 *Department of Neurology, School of Medicine, Zanjan University of Medical Sciences (ZUMS), Zanjan, Iran.*; 4 *School of Mathematics, Institute for Research in Fundamental Sciences (IPM), Tehran, Iran.*; 5 *Experimental Laboratory Immunology, Department of Microbiology and Immunology, KU Leuven, Leuven, Belgium.*; 6 *Specialized Immunology Laboratory of Dr. Shahrooei, Ahvaz, Iran.*; 7 *Cellular, Molecular and Genetics Research Center, Isfahan University of Medical Sciences, Isfahan, Iran.*; 8 *Department of Genetics, Medical School, Isfahan University of Medical Sciences, Isfahan, Iran.*

**Keywords:** BSCL2, GJB1, whole exome sequencing, Iranian Charcot-Marie-Tooth patients

## Abstract

Charcot-Marie-Tooth disease (CMT) is the most common hereditary neuropathy of the peripheral nervous system with a wide range of severity and age of onset. CMT patients share similar phenotypes which make it often impossible to identify the disease types based on clinical presentation and electrophysiological studies alone. In recent years, novel genetic diagnostic approaches such as whole exome sequencing (WES) has provided a ground for accurate diagnosis of CMT through identification of the disease-causing mutation(s). In the present study, that approach was effectively employed. Two unrelated large pedigrees with multiple affected cases of various pattern of inheritance (one autosomal dominant and one X-linked) were included. Clinical and electrophysiological data were obtained. DNA sample from each pedigree’s proband was subjected to WES. Data analysis was performed using an in-house developed pipeline, adopted from GATK and ANNOVAR. Candidate variant segregation was evaluated by PCR-based Sanger sequencing. A known but extremely rare (unreported in the Middle Easterners) mutation in *BSCL2* (c.C269T:p.S90L) as well as a novel hemizygous variant in *GJB1* (c.G224C:p.R75P) were identified and segregations were confirmed by Sanger sequencing. This study supports effectiveness of WES for genetic diagnosis of CMT in undiagnosed families.

Charcot-Marie-Tooth disease (CMT), charact-erized by the slow progressive weakness, impaired exteroception, distal muscular atrophy as well as foot deformity is a heterogeneous neuromuscular disease with a prevalence of nearly 1 in 2,500 in western countries (1).

Diverse age of onset and clinical severity among CMT patients even within the same family are important hallmarks of the disease (2). Based on the median motor nerve conduction velocity (MNCV), CMT is majorly divided into three subtypes: a demyelinating form (CMT1) with MNCV of less than 38 m/s, an axonal form (CMT2) with MNCV higher than 38 m/s or normal and an intermediate form with MNCV about 25-45 m/s. Nevertheless, patients of different subtypes share similar phenotypic traits, making differentiation of the disease type, solely based on the clinical and electrophysiological data, quite challenging. Com-bining genetic information with clinical characteris-tics provides a better understanding of the disease subtype, though extracting accurate genetic infor-mation using conventional methods has proven challenging (3,4).

CMT causative mutations displaying various frequencies in various populations have been identi-fied in over 80 genes (5), driving diverse patterns of inheritance in familial CMT including autosomal dominant (AD), autosomal recessive (AR), and dominant or recessive X-linked (3). Considering the high rate of consanguineous marriages in some communities such as the Mediterranean and Middle Easterners, the autosomal recessive form of CMT is presumably more prevalent. Published records have estimated occurrence of AR-CMT in 30 to 50 % of all cases diagnosed in these populations (1, 6, 7), while AD forms of CMT are dominant in Western Europe, the United States and Japan (8). However, there is little comprehensive and precise informa-tion on the frequency of genetic subtypes and their phenotypic correlations in patients suffering from CMT within some populations including Iranians.

Remarkably, genetic diagnosis of CMT is admittedly a big challenge. Diagnostic approaches for a long time have relied on conventional methods such as multiplex ligation-dependent probe amplifi-cation (MLPA) or PCR-based Sanger sequencing, leaving numerous patients undiagn-osed. Since 2011, whole exome sequencing (WES) has been introduced as an efficient tool for diagnosis of known and novel mutations associated with CMT in patients for whom, conventional methods have failed to determine genetic causes of the disease (9, 10). In this study, WES was employed to find the genetic causes of CMT in two distinct pedigrees, manifesting an AD or X-linked type of CMT, which had remained genetically undiagnosed with either MLPA or Sanger sequencing.

## Materials and Methods

**Clinical evaluation**

The study was conducted in accordance with the Declaration of Helsinki, and approved by Zanjan University of Medical Sciences Ethics Committee (ZUMS.REC.1395.145). Before initia-tion of the study, written and informed consent was obtained from all participants for their participation in the study and for publication of this report. Parental consent was obtained for all participants under the age of 18.

Nerve conduction studies and electromy-ography (using MEB-9402; Nihon Kohden, Tokyo, Japan) were performed at the neuroelectrodia-gnostic clinics of Vali-e-Asr university Hospita (Zanjan University of Medical Sciences).

**Whole exome sequencing**

**Fig. 1:**
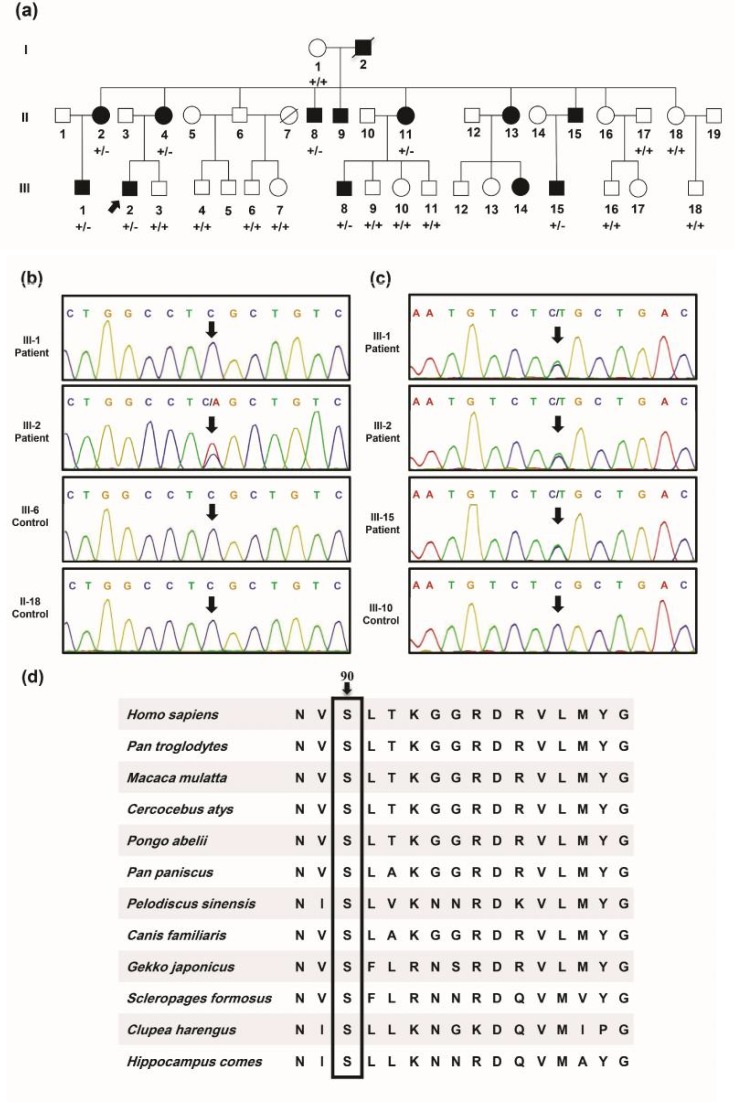
Pedigree, sequencing chromatograms, and conservation analysis in a family affected from BSCL2 mutation. a: pedigree of family 1 having autosomal dominant form of CMT disease is drawn. A heterozygous mutation in the BSCL2 gene was identified in proband III-2 (pointed with an arrow), II-2, II-4, II-8, II-11, III-1, III-8, and III-15 members of the pedigree. +/+: WT, wild type; +/-: heterozygous for the mutation. b: chromatograms of the heterozygous c.C1049A (S350X) variant in exon 7 of ACTA1 are illustrated. Arrows are pointing to the mutated nucleotide position in the patients. c: chromatograms of the heterozygous c.C269T (S90L) mutation in exon 3 of BSCL2 are illustrated. Arrows are pointing to the mutated nucleotide position in the patients. d: amino acid alignment of seipin protein orthologs from several species using Clustal Omega is presented. Arrow head is pointing to the evolutionary conserved amino acid which is mutated in studied patients

**Fig. 2:**
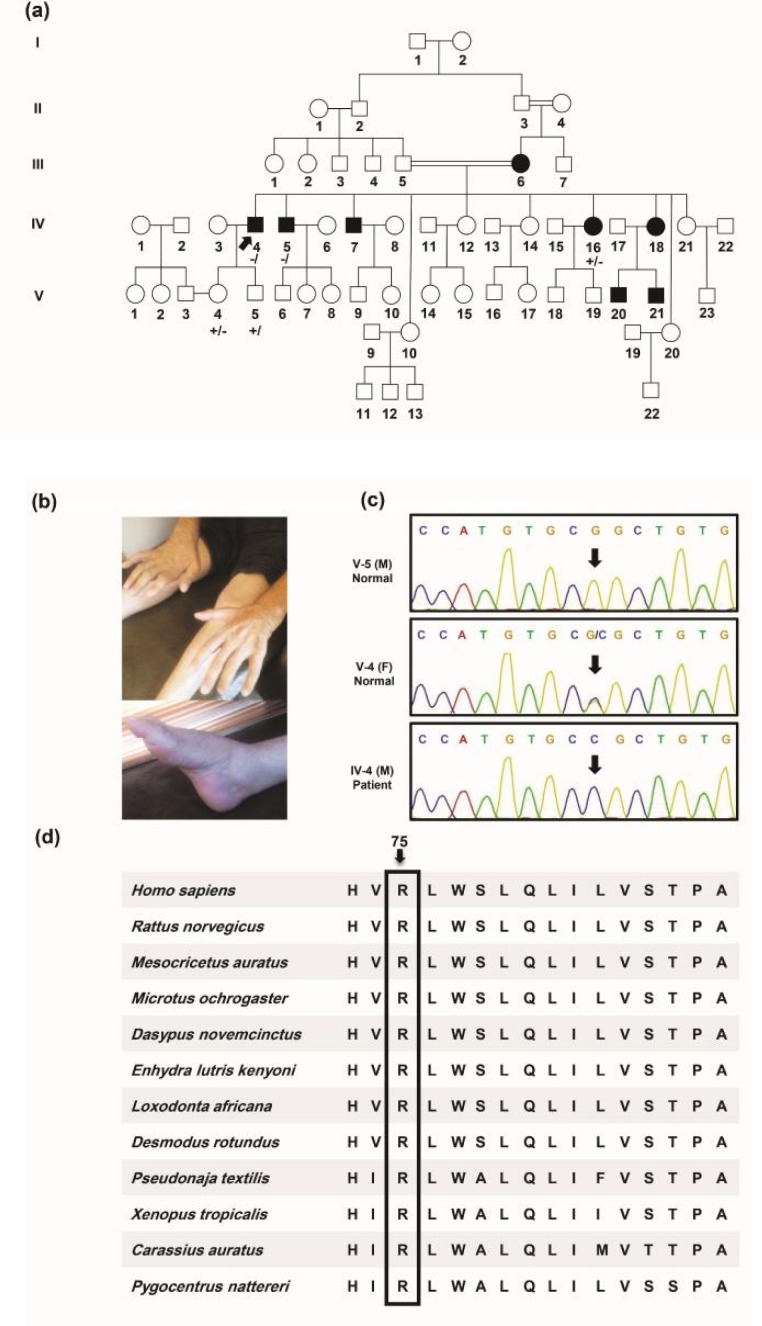
Pedigree, photographs of the proband’s (IV-4) deformities of the feet and hands, sequencing chromatograms, and conservation analysis of the mutated amino acid in a family with c.G224C:p.R75P variant. a: Pedigree of the family 2 with X-linked dominant Charcot-Marie-Tooth. A hemizygous exon2:c.G224C:p.R75P variant in the GJB1 gene was identified in proband IV-4 (pointed with an arrow). Genotype of the GJB1 variant is indicated under each person examined (-/: male hemizygous for the variant; +/-: female heterozygous for the variant; +/: male negative for the variant). b: feet and hands deformities in the proband (IV-4). c: sequencing chromatograms of a healthy male, a healthy female heterozygous for c.G224C:p.R75P variant, and an affected male for the same variant. d: evaluation of amino acid evolutionary conservation using Clustal Omega. As illustrated, the mutation site is highly conserved in various species

From each study subject, genomic DNA was extracted from whole peripheral blood utilizing innuPREP Blood DNA Mini Kit (Analytika Jena, Germany) according to the manufacturer’s instruction. DNA samples from each pedigree’s proband (Figure 1a, III-2 in family 1; Figure 2a, IV-4 in family 2) were then subjected to WES at Macrogen (Seoul, South Korea) on the Genome Analyzer HiSeq 4000 (Illumina, San Diego, CA, USA, 101-bp paired- end reads). The library had been prepared using SureSelect XT Library Prep Kit (Agilent Technologies, CA, USA). Data analysis was performed using an in-house developed pipeline, adopted from GATK and ANNOVAR (11, 12). 

**PCR-based Sanger sequencing**

Candidate variant segregation from exome data was evaluated by PCR-based Sanger sequencing on an ABI 3500 Genetic Analyzer (Applied Biosystems, Foster City, CA, USA). The specific primers were designed using Geneious software. Primers are as follows: *ACTA1*_fwd 5-CACGATGTACCCTGGGA-3, *ACTA1*_rev 5′-AAAGAAAGTGACTGCGG-3′; *BSCL2*_fwd 5-AGGGTGCCTGTTCTGAGAGA-3, *BSCL2*_rev 5′- CTGGTCTCGAACTCCCAACC-3′ and *GJB1*_ fwd 5-ATGGCTCTCGGTCATCTTCATCTTC-3,* GJB1*_rev. 5′-CATGAAGACGGCCTCAAACAAC AG-3′.

## Results

**Family 1**

The family pedigree is displayed in Figure 1a. Twenty members of the pedigree including 8 affected and 12 unaffected individuals who consented to this study are indicated. In 7 out of 8 patients, symptoms emerged around the ages of 5 to 7, and at the age of 10 for one patient (III-1), with distal muscle weakness, gait difficulties with progressive appearance of foot drop, hammer toe, and claw hands, as the most pronounced phenotypes. Clinical features of three patients are summarized in Table 1.

The proband (III-2) is a 19-year-old boy suffering from unsteady gait and frequent falling started at the age of 7; yet, he was able to walk independently. Neurological examination at the age of 19 revealed a steppage gait with mild spastic feature, thenar atrophy, scoliosis, foot deformities, equinovarus as well as muscular atrophy and weakness in the lower and upper limbs, which showed predominantly distal involvement. Electrophysiological features of the proband were consistent with an axonal neuropathy (Table 1).

To identify genetic cause of the disease, screening for the prevalent CMT-causing variations i.e. peripheral myelin protein 22 (*PMP22*; OMIM 601097), duplication and point mutations in myelin protein zero (*MPZ*; OMIM 159440), and mitofusin 2 (*MFN2*; OMIM 608507) genes, had been performed using MLPA and PCR-based Sanger sequencing, respectively. Once the screening appeared negative, WES was implemented on proband’s genome. Initial analysis have unraveled a novel stop-gain variant (c.C1049A:p.S350X) in actin alpha 1, skeletal muscle (*ACTA1*; OMIM 102610) gene, and a known but extremely rare c.C269T:p.S90L mutation in Bernardinelli-Seip congenital lipodystrophy type 2 (*BSCL2*; OMIM 606158) gene. There are no reports regarding allele frequency of *BSCL2* (c.C269T:p.S90L) variant in various databases such as the 1000- Genome Project, ESP6500, ExAC, CG69, and Iranome project.

While Sanger sequencing have confirmed presence of the p.S350X variant in *ACTA1* gene in the proband, other pedigree members who were studied, including the patients and healthy individuals lacked the variant (Figure 1b). On the contrary, the p.S90L variant in the *BSCL2* gene was detected in all the affected members, unlike the unaffected members of the family (Figure 1c). The amino acid residue 90 of the human seipin protein is highly conserved among vertebrates (Figure 1d).

**Family 2**

A five-generation pedigree, depicted in Figure 2a, is presented as the second family with five members, 3 males and 2 females, suffering from an apparently X-linked dominant CMT. No male-to-male transmission was observed in the pedigree and the phenotype was more severe in males compared to females.

**Table 1:** Clinical features in four affected members with neurophysiologic characteristics of family 1 and family 2’s probands

**Family 2 with ** ***GJB1*** ** c.G224C:p.R75P variant**		**Family 1 with ** ***BSCL2*** ** c.C269T:p.S90L mutation**		**Characteristics**
**proband** **IV-4**	**proband** **III-2**	**III-15**	**III-1**	**Patients**
Male	Male	Male	Male	Gender
21	7	7	10	Age at onset (year)
22	19	14	12	Age at the first visit (year)
CMT	CMT	CMT	CMT	Clinical diagnosis
walking difficulty	Unsteady gait frequent falls	Unsteady gait frequent falls	Unsteady gait toe walking frequent falls	Presenting symptom
Steppage gait	Steppage gait with mild spastic feature	Steppage gait	Steppage gait	Gait
Absent tendon reflexes in both upper and lower extremities, absent tendon reflexes with weakness of foot dorsiflexion at the ankle	UL: + LL: +++	UL: ++ LL: ++	UL: ++ LL: +++	DTR
Weakness of the feet and ankles, symmetrical atrophy of muscles below the knee (stork leg appearance), atrophy of intrinsic hand muscles, predominantly thenar	Distal> proximal both UL&LL asymmetric calf atrophy thenar atrophy	Distal = proximal both UL&LL pretibial atrophy	Distal> proximal both UL&LL thenar atrophy pretibial atrophy	Muscular atrophy and weakness
Bilateral foot drop, pes cavus, hammer toe	Pes cavus hammer toe	Bilateral foot drop pes cavus	Foot drop pes cavus	Foot deformity
Equinovarus, arthralgia, mild to moderate sensory deficits of position, vibration and pain in the feet, claw hands	LL hyperreflexia scoliosis, equinovarus, dermatographism, reduced gag reflex	Double Babinski sign stridor, previous tendon release of achilles, scoliosis, equinovarus	LL hyperreflexia claw hands, delayed wound healing, equinovarus, scoliosis	Additional features
Absent	5.9	NT	NT	Median CMAP (mV)
Absent	54.4	NT	NT	Median MNCV (m/s)
Absent	4.4	NT	NT	Median SNAP (µV)
Absent	34.6	NT	NT	Median SNCV (m/s)
2.25 (mV)(normal: 6 mV)	2.6	NT	NT	Ulnar CMAP (mV)
20 (m/s) (normal: 50 m/s)	49.8	NT	NT	Ulnar MNCV(m/s)
Absent	4.3	NT	NT	Ulnar SNAP (µV)
Absent	28.1	NT	NT	Ulnar SNCV (m/s)
Absent	0.253	NT	NT	Tibial CMAP (mV)
Absent	37	NT	NT	Tibial MNCV (m/s)
Absent	Absent	NT	NT	Peroneal CMAP (mV)
NT	Absent	NT	NT	Sural SNAP (µV)
Absent	NT	NT	NT	Radial SNAP (µV)

CMT: Charcot-Marie-Tooth disease; +: hyporeflexia; ++: normal; +++: hyperreflexia; UL: upper limb; LL: lower limb; MNCV: motor nerve conduction velocity; CMAP: compound muscle action potential; SNCV: sensory nerve conduction velocity; SNAP: sensory nerve action potential; DTR: deep tendon reflexes; NT: not tested.

In terms of age, the onset of the disease ranged from 18 to 23 years in all patients excluding V-20 and V-21, whose initial symptoms appeared at around 5 years of age.

The proband (IV-4) was a 63-year-old man who had been encountered with gait problem at the age of 21. He was not wheelchair-bound as though he was able to walk using one crutch. At the age of 63, a neurological examination revealed distal muscle weakness and atrophy with sparing of proximal limb, bilateral foot drop, pes cavus, hammer toe and claw hands (Figure 2b). His deep tendon reflexes were absent in all extremities. There was no central nervous system involvement including extensor plantar responses and dysarthria. He exhibited no symptoms or signs of optic nerve atrophy, hip dysplasia, hearing loss, dexterity problems, difficulty balance, scoliosis and burning feet. Furthermore, no record of foot surgery was found.

Nerve conduction studies of median, ulnar, peroneal, tibial, and radial nerves in the proband demonstrated a demyelinating hereditary sensory-motor polyneuropathy. Motor-nerve-conduction studies revealed low amplitude compound muscle action potential only from the left ulnar nerve, while no sensory motor responses were recorded from other nerves. The clinical details and electrophysiological ﬁndings of the proband are summarized in Table 1.

With regards to the other family members, a less severe phenotype of foot and hand deformities was reported in proband’s mother (III-6). Unfortunately, neither she nor some other affected members (IV-7, IV-18, V-20, and V-21) have agreed to participate to this study. Genetic cause of CMT in this family was identified using WES on the proband’s genomic DNA (IV-4). The analysis unraveled the presence of a novel hemizygous variant (c.G224C:p.R75P) in gap junction protein, beta1 (*GJB1*; OMIM 304040).

Sanger sequencing revealed co-segregation in this family (Figure 2c). The affected proband was hemizygous, whereas his unaffected daughter (V-4) was heterozygous for this locus, and his unaffected son (V-5) lacked the variant. The affected brother (IV-5) was hemizygous, and the affected sister (IV-16) was heterozygous for the variant. No symptoms of the disease have been witnessed in her sons thus far.

*GJB1* encodes Cx32, a member of the connexin family, or gap junction proteins. Position 75 in *GJB1* is highly conserved across all connexins (Figure 2d).

## Discussion

Based on WES data, segregation and genotype-phenotye correlation study, the identified variations in *BSCL2* and *GJB1* are the causes of type 2 and X-linked dominant 1 CMT neuropathies in the first and second studied families, respectively.

The identified c.C269T:p.S90L mutation in our study is a particularly rare mutation and of low frequency in CMT2 (18). To our knowledge, this mutation has only been reported in Europeans (14, 19-22), and Taiwanese (18), so far. The present study is the first report of the c.C269T:p.S90L mutation identified in Iranian patients affected with CMT2

Seipin is a pivotal integral membrane protein of endoplasmic reticulum (ER), ubiquitously expressed in all cells and in particular, brain nerve cells (23). Gain of function mutations in BSCL2 such as c.A263G:p.N88S and c.C269T:p.S90L are associated with both upper and lower motor neuron disruptions (15). The effect mechanisms of those mutations are partially described. 

The c.C269T:p.S90L mutation in the N-glyco-sylation site of seipin alters protein structure and induces misfolding which leads to increased ubiquitination and degradation of seipin through ubiquitin–proteasome system (UPS). Subsequently, misfolded proteins accumulate in the ER, resulting in endoplasmic reticulum stress-mediated cell death (24). 

Besides CMT2 (25), heterozygous mutations within *BSCL2 *have been associated to a wide range of phenotypic abnormalities such as Silver syndrome/spastic paraplegia 17 and distal hereditary motor neuropathy type V (dHMNV). The CMT2 phenotype itself is strongly heterogeneous and presents a variable penetrance. Conventional understanding is that genetic diagnosis of CMT2 could not be attained in 75% of the clinically diagnosed cases of CMT2 using common diagnostic methods (26). For a robust genetic diagnosis of CMT2 as well as CMT2 differentiation from Silver syndrome and dHMNV, seipin/*BSCL2* mutation screening seems to be important, particularly in patients with apparently upper motor neuron and lower motor neuron involvement. Subsequently, clinicians would be able to make correlation between clinical phenotypes and molecular results. 

In the second studied family, an X-linked dominant 1 CMT (CMTX1) was confirmed. CMTX1 is known as the 2nd most common form of hereditary motor and sensory neuropathy which constitutes roughly 90% of the entire X-linked CMTs (27). WES successfully detected a novel hemizygous missense variant (c.G224C:p.R75P) in *GJB1*, which co-segregated with the disease within the pedigree. 

Cx32, is widely expressed in myelinating Schwann cells within the peripheral nervous system. Gap junction proteins are typically assembled into connexons (hemichannels) in the Golgi apparatus, forming the gap junction channels, which promotes the transference of small molecules between the adjacent cells, or layers of the myelin sheath. Over 400 mutations have been identified throughout the *GJB1.* The mutations often result in loss-of-function than gain-of-function at the protein level (27). In this study, we identified the novel c.G224C:p.R75P variant in* GJB1*. Arginine in the position 75 of Cx32 is highly conserved amongst all the members of the connexin family (28). The truncated connexin 32 does not assemble in a proper manner and entraps in the Golgi. Although the Golgi-retained truncated protein could be degraded by lysosomal proteolysis or be transferred from the Golgi to the ER to be degraded by proteasomes (29, 30). The absence of functional protein at plasma membrane of peripheral nerves results in neuropathy. 

Accurate mutational diagnosis of CMT is extremely important for genetic counseling and even prenatal diagnosis (2). Remarkably, plenty of rare genes/potential mutations are not being tested for everyone due to the costliness of conventional genetic testing which initially starts with genetic analysis of *PMP22*,* GJB1*,* MPZ*, and* MFN2*, world-wide (31). As a result, almost 50 percent of the patients remain genetically undiagnosed (26). Moreover, recently exome-first approach has been used for precise diagnosis of phenotypic and genetic heterogeneous diseases (32). Here we declare the diagnostic utility and affordability of WES not only for the patients whom initial genetic screening by conventional methods has been negative, but also as a first approach for genetic testing of such a diverse and complex trait. 
